# Determination of the Accuracy of Computed Tomography in Staging Primary Rectal Carcinoma and Lymph Node Spread Post Chemoradiation Therapy

**DOI:** 10.7759/cureus.105371

**Published:** 2026-03-17

**Authors:** Varsha Ganesh Babu, Vivek B Badiger, Vinay M D Prabhu, Ritika Agarwal, Umesh Krishnamurthy

**Affiliations:** 1 Department of Radiology, Shri Atal Bihari Vajpayee Medical College and Research Institute, Bengaluru, IND; 2 Department of Radiology, MS Ramaiah Medical College, Bengaluru, IND

**Keywords:** computed tomography, diagnostic accuracy, histopathology, lymph node involvement, neoadjuvant chemoradiation, rectal carcinoma, staging

## Abstract

Objective: This study aims to evaluate the diagnostic accuracy of multidetector computed tomography (MDCT) in post-chemoradiation restaging of rectal carcinoma. The primary endpoints were T (tumor) and N (nodal) staging accuracy using surgical histopathology as the reference standard. Diagnostic accuracy was defined in terms of sensitivity, specificity, positive predictive value, negative predictive value, and overall concordance. A secondary objective was to assess the role of MDCT in detecting distant metastasis in correlation with intraoperative and available clinical findings.

Methodology: A prospective analytical observational study was conducted in the Department of Radiodiagnosis at M. S. Ramaiah Hospitals, Bengaluru, from November 2018 to June 2020. Twenty-seven patients with biopsy-proven rectal carcinoma underwent CT imaging of the abdomen and pelvis following chemoradiation and before surgical resection. MDCT-based T (tumor), N (nodal), and M (metastasis) staging were compared with intraoperative assessments and histopathological results. Diagnostic performance was evaluated by calculating sensitivity, specificity, positive predictive value, negative predictive value, and overall accuracy.

Results: A total of 27 patients were analyzed, including 16 (59.3%) females and 11 (40.7%) males, with most aged 51-70 years. Bleeding per rectum was the most common symptom in 26 (96.3%) patients, followed by blood in stools in 24 (88.9%) and anemia in 21 (77.8%). Tumors were primarily located in the rectum among 21 (77.8%) patients, and were predominantly adenocarcinomas in 21 (77.8%) patients. Perirectal fat involvement was observed in 18 (66.7%) patients, and regional lymph node invasion in eight (29.6%).

Conclusion: MDCT is a reliable modality for staging rectal cancer, particularly for advanced stages (T3-T4), demonstrating high accuracy in assessing tumor extent, nodal involvement, and distant metastases. Its sensitivity for early-stage lesions remains lower than that of MRI, highlighting the complementary role of multimodal imaging in preoperative evaluation.

## Introduction

Colorectal cancer is among the most common malignancies worldwide, with rectal tumors representing a substantial proportion of cases in both the United States and the United Kingdom [1,2]. In the United States, approximately one-third of colorectal cancers originate in the rectum [1]. Large multicenter trials in the United Kingdom, such as FOxTROT and MERCURY, have highlighted the importance of accurate staging in guiding neoadjuvant treatment decisions [2,3]. Unlike colon cancer, where adjuvant chemotherapy is standard for stage III disease, rectal cancer often requires neoadjuvant chemoradiation, followed by surgery, particularly for locally advanced tumors [4]. Thus, precise staging before treatment and reliable restaging after chemoradiation are essential for selecting therapy, informing prognosis, and planning surgical strategy [5].

Magnetic resonance imaging (MRI) and endorectal ultrasound (ERUS) are established methods for local staging of rectal cancer [4,5]. Computed tomography (CT), however, remains widely used to detect distant metastases and to assist in regional and nodal assessment, particularly in settings with limited access to advanced imaging [6]. CT-based nodal evaluation relies primarily on size criteria, which may underestimate disease when micrometastases exist in normal-sized nodes and overestimate it in cases of reactive enlargement [6,7]. These limitations are especially relevant after chemoradiation therapy (CRT), when accurate assessment of tumor regression and nodal downstaging is critical for determining surgical resectability and identifying candidates for organ-preserving approaches.

This study aims to evaluate the diagnostic accuracy of CT for staging primary rectal tumors and lymph node metastases after CRT, and to correlate imaging findings with surgical and histopathological outcomes. While MRI and ERUS are well-established in this context, limited evidence exists regarding the reliability of CT post CRT. Addressing this gap is particularly important given CT’s widespread use in routine practice, especially in resource-constrained environments. By systematically assessing CT’s diagnostic performance, this study seeks to clarify its role in rectal cancer restaging and support evidence-based clinical decision-making.

The primary objective of this study was to evaluate the diagnostic accuracy of multidetector computed tomography (MDCT) in post-chemoradiation restaging of rectal carcinoma, specifically for T (tumor) and N (nodal) staging, using surgical histopathology as the reference standard. Diagnostic accuracy was defined as sensitivity, specificity, positive predictive value (PPV), negative predictive value (NPV), overall accuracy, and concordance between MDCT findings and histopathological staging. A secondary objective was to assess the role of MDCT in detecting distant metastasis (M staging). For M staging, radiological findings were correlated with intraoperative findings and available clinical or imaging records, as histopathology of the resected rectal specimen does not routinely determine distant metastatic status unless metastatic lesions are sampled.

## Materials and methods

Study design and setting

This prospective, analytical, observational study was conducted in the Department of Radiodiagnosis at M. S. Ramaiah Hospitals, Bengaluru, between November 2018 and June 2020. The study evaluated the diagnostic accuracy of MDCT in restaging biopsy-proven rectal carcinoma after completion of neoadjuvant CRT, using surgical findings and histopathological examination as the reference standard.

Eligibility criteria

Patients were included if they had histologically confirmed primary rectal carcinoma, had completed standard long-course neoadjuvant CRT, underwent contrast-enhanced CT (CECT) of the abdomen and pelvis for restaging, and subsequently proceeded to definitive surgical resection with the availability of complete histopathological TNM staging.

Patients were excluded if they had undergone prior rectal surgery, had recurrent rectal carcinoma, had incomplete CRT, had distant metastases detected before initiation of CRT, had inadequate or poor-quality CT imaging, or did not proceed to surgical resection.

Sample size

Based on Chiesura-Corona et al. [8], who reported CT sensitivities of 81.9% for Tis-T2, 82.5% for T3, and 75% for T4, a minimum sample size of 25 patients was calculated at a 95% confidence level and 15% relative precision.

Data collection and imaging protocol

All eligible patients presenting for CT during the study period were recruited after obtaining written informed consent. Clinical and demographic data were recorded using a structured proforma. To standardize post-treatment response assessment, CT imaging was performed six to eight weeks after completion of CRT in accordance with institutional oncologic protocol. Surgical resection was performed within one to three weeks following CT acquisition. The interval between CRT completion and CT examination was recorded in weeks, and the interval between CT and surgery was recorded in days. This standardization was intended to minimize variability in tumor regression and staging accuracy in the post-CRT setting. CT examinations were performed using either a Toshiba Asteion VP single-helical CT scanner (Toshiba, Tokyo, Japan) or a Siemens Somatom 128-slice multidetector CT scanner (Siemens Healthineers, Forchheim, Germany). Serial axial images were obtained from the dome of the diaphragm to the pubic symphysis. For reproducibility, CT acquisition parameters were standardized as follows: images were acquired at a slice thickness of 5 mm and reconstructed at 1-2 mm thickness for detailed multiplanar assessment. Intravenous non-ionic iodinated contrast was administered at a dose of approximately 1.5 mL/kg (total volume of 80-120 mL) using a power injector at a rate of 3-4 mL/sec. Imaging was performed in the portal venous phase (70-80 seconds after contrast injection). No routine arterial phase imaging was performed.

Patients fasted for at least six hours prior to imaging. Oral contrast (800-1000 mL diluted iodinated contrast) was administered for bowel opacification. Routine rectal contrast administration or rectal distension was not performed. Additional bowel preparation was not standardized, and antispasmodic agents were not routinely administered.

Multiplanar reformatted (MPR) images in sagittal and coronal planes were systematically generated and reviewed in all cases to improve local tumor evaluation, assessment of mesorectal extension, and adjacent organ involvement. CT evaluation included TNM staging, tumor location, perirectal fat stranding, distance from the anal verge, invasion of adjacent organs, and nodal status.

Image interpretation and staging criteria

All CT images were independently reviewed by two radiologists with more than five years of experience in abdominal imaging. The radiologists were blinded to histopathological results and intraoperative findings at the time of CT interpretation, although they were aware that patients had received CRT. Discrepancies were resolved by consensus. Interobserver agreement was assessed using Cohen’s kappa coefficient.

T-staging criteria (post chemoradiation)

Tumor staging was assigned according to TNM criteria adapted for post-CRT evaluation. Lesions confined to the rectal wall without extension beyond the muscularis propria were classified as T1-T2. Tumors demonstrating extension beyond the muscularis propria into the perirectal fat were classified as T3. The T4 stage was assigned when there was a clear invasion of adjacent organs or structures.

In the post-CRT setting, smooth linear or homogeneous fibrotic stranding in the perirectal fat without nodularity was interpreted cautiously as treatment-related fibrosis. In contrast, nodular, irregular, or mass-like soft tissue extension beyond the rectal wall was considered indicative of residual viable tumor infiltration and categorized as T3.

Nodal staging criteria

Lymph node assessment was performed using both size and morphological characteristics. Short-axis diameter measurement was used, and nodes with a short-axis diameter ≥5 mm were considered suspicious. In addition to size, morphological features, including round configuration, irregular or spiculated margins, central necrosis, and heterogeneous enhancement, were considered suggestive of malignant involvement. Regional nodal stations evaluated included mesorectal, superior rectal, presacral, internal iliac, and obturator lymph nodes within the scan field.

Size criteria were not used in isolation due to recognized limitations in the post-CRT setting, where reactive or fibrotic changes may alter nodal size. It is acknowledged that CT has inherent limitations in differentiating benign from malignant nodes, particularly in cases of micrometastases within normal-sized nodes or fibrotic treated nodes.

Distant metastasis assessment

Distant metastasis (M staging) was assessed by evaluating the liver, lung bases included within the scan range, peritoneum, and distant lymph nodes.

Histopathology of the resected specimen was considered the reference standard for T and N staging. For M staging, correlation was performed using intraoperative findings and available clinical and imaging records, as routine surgical histopathology does not determine distant metastatic status unless metastatic lesions are biopsied or resected.

Pathologists were blinded to the CT staging results to minimize observer bias. In cases where CT findings were equivocal, staging was assigned based on consensus interpretation between the two radiologists. If definitive categorization was not possible, cases were labeled as Tx or Nx. Such cases were included in descriptive analysis but excluded from sensitivity and specificity calculations to avoid distortion of diagnostic accuracy measures.

Statistical analysis

Data entry was performed in Microsoft Excel (Microsoft Corporation, Redmond, WA), and statistical analyses were conducted using IBM SPSS Statistics for Windows, version 22.0 (IBM Corp., Armonk, NY). Categorical variables were summarized as frequencies and percentages, and continuous variables were expressed as mean ± standard deviation. Associations between categorical variables were assessed using the chi-square test. Diagnostic performance parameters, including sensitivity, specificity, PPV, NPV, and overall diagnostic accuracy, were calculated with corresponding 95% confidence intervals (CI). Interobserver agreement was assessed using Cohen’s kappa coefficient and interpreted as poor (<0.20), fair (0.21-0.40), moderate (0.41-0.60), substantial (0.61-0.80), or excellent (>0.80). A p-value of <0.05 was considered statistically significant.

Ethical consideration

The study was approved by the Institutional Ethics Committee of M. S. Ramaiah Hospitals, Ramaiah Medical College, under approval number EC/PG-66/2018. Written informed consent was obtained from all participants before enrollment. All procedures were conducted in accordance with institutional ethical guidelines and the principles of the Declaration of Helsinki (1964) and its later amendments.

## Results

A total of 27 patients were analyzed. The majority were aged 51-70 years (16, 59.3%), while five (18.5%) were <40 years and four (14.8%) were >70 years. Females comprised 16 (59.3%) of the cohort, and 11 (40.7%) were males. A mixed dietary pattern was reported by 20 (74.1%) patients. The most frequent duration of symptoms was three months in 10 (37.0%) patients, followed by six months in six (22.2%) patients. Abdominoperineal resection (APR) was the most commonly performed surgical procedure in 14 (51.9%) patients, followed by low anterior resection (LAR) in 10 (37.0%) (Table 1).

**Table 1:** Baseline demographic and clinical characteristics of the study population (N = 27).

Variables	Total number, N (%)
Age	
<40 years	5 (18.5%)
41 to 50 years	4 (14.8%)
51 to 60 years	6 (29.6%)
61 to 70 years	8 (29.6%)
>70 years	4 (14.8%)
Sex	
Male	11 (40.7%)
Female	16 (59.3%)
Diet	
Mixed	20 (74.1%)
Vegetarian	7 (25.9%)
Duration	
1 month	1 (3.7%)
2 months	3 (11.1%)
3 months	10 (37%)
4 months	2 (7.4%)
5 months	2 (7.4%)
6 months	6 (22.2%)
7 months	1 (3.7%)
9 months	1 (3.7%)
12 months	1 (3.7%)
Surgery type	
Abdominoperineal resection (APR)	14 (51.9%)
Low anterior resection (LAR)	10 (37%)
Pelvic exenteration	1 (3.7%)
Posterior pelvic exenteration (PPE)	1 (3.7%)
Proctocolectomy	1 (3.7%)

Among the 27 patients, 13 (48.1%) reported smoking or chewing habits and alcohol consumption. Bleeding per rectum was present in 26 (96.3%) patients, pain in the rectum in 10 (37.0%), and abdominal pain in 15 (55.6%). Weight loss was observed in 18 (66.7%) patients, anemia in 21 (77.8%), diarrhea in 12 (44.4%), constipation in four (14.8%), and blood in stool in 24 (88.9%). On CT evaluation, perirectal fat involvement was seen in 18 (66.7%) patients, adjacent organ invasion in two (7.4%), lymph node involvement in six (22.2%), and no pelvic lymphadenopathy was observed in any patient (100%) (Table 2).

**Table 2:** Clinical symptoms and CT findings of the study population (N = 27).

Variables	No	Yes
	Total number, N (%)	Total number, N (%)
Symptoms		
Smoking/chewing	14 (51.9%)	13 (48.1%)
Alcohol	14 (51.9%)	13 (48.1%)
Bleeding per rectum	1 (3.7%)	26 (96.3%)
Pain in the rectum	17 (63%)	10 (37.%)
Abdominal pain	12 (44.4%)	15 (55.6%)
Weight loss	9 (33.3%)	18 (66.7%)
Anemia	6 (22.2%)	21 (77.8%)
Diarrhea	15 (55.6%)	12 (44.4%)
Constipation	23 (85.2%)	4 (14.8%)
Blood stools (hematochezia)	3 (11.1%)	24 (88.9%)
CT findings		
Perirectal fat	9 (33.3%)	18 (66.7%)
Adjacent organ	25 (92.6%)	2 (7.4%)
Lymph nodes	21 (77.8%)	6 (22.2%)
Pelvic lymph node	27 (100%)	0 (0%)

Most tumors were located in the rectum (21, 77.8%), with two (7.4%) in the lower rectum, two (7.4%) in the upper rectum, and one (3.7%) each in the mid-rectum and rectosigmoid region. Adenocarcinoma was the predominant histologic type in 21 (77.8%) patients, followed by mucinous adenocarcinoma in two (7.4%) and signet ring cell carcinoma in one (3.7%). Histologic grading revealed 19 (70.4%) moderately differentiated, three (11.1%) well-differentiated, and three (11.1%) poorly differentiated tumors. Regarding the extent of invasion, the muscularis propria was most frequently involved in 13 (44.4%) patients, followed by the subserosa in seven (25.9%), the submucosa in three (11.1%), and adjacent organ invasion in one (3.7%) (Table 3).

**Table 3:** Tumor characteristics and histopathological features of the study population (N = 27).

Parameters	Total number, N (%)
Tumor size	
Lower rectum	2 (7.4%)
Mid rectum	1 (3.7%)
Recto sigmoid	1 (3.7%)
Rectum (site not further specified)	21 (77.8%)
Upper rectum	2 (7.4%)
Histologic type	
Adenocarcinoma	21 (77.8%)
Mucinous adenocarcinoma	2 (7.4%)
Mucinous carcinoma	1 (3.7%)
No	2 (7.4%)
Signet ring cell	1 (3.7%)
Histologic grade	
No	2 (7.4%)
Well differentiated	3 (11.1%)
Moderately differentiated	19 (70.4%)
Poorly differentiated	3 (11.1%)
Extent	
Adjacent organ	1 (3.7%)
Muscularis propria	13 (44.4%)
No	3 (11.1%)
Submucosa	3 (11.1%)
Subserosa	7 (25.9%)

Of the 27 patients, eight (29.6%) had regional lymph node invasion, six (22.2%) had lymphatic invasion, three (11.1%) had perineural invasion, and two (7.4%) had venous invasion (Table 4).

**Table 4:** Metastasis among subjects.

Parameters	Absent	Present
	Total number, N (%)	Total number, N (%)
Regional lymph node invasion	19 (70.4%)	8 (29.6%)
Venous invasion	25 (92.6%)	2 (7.4%)
Perineural invasion	24 (88.9%)	3 (11.1%)
Lymphatic invasion	21 (77.8%)	6 (22.2%)

Tumor regression scores demonstrated that 12 (44.4%) patients had a score of three, seven (25.9%) had a score of one, five (18.5%) had a score of two, and two (7.4%) had a score of zero. Only one (3.7%) patient showed a regression score of four (Table 5).

**Table 5:** Regression score.

Parameters		Count	%
Regression score	Zero	2	7.4%
	One	7	25.9%
	Three	12	44.4%
	Two	5	18.5%
	Four	1	3.7%

Table 6 demonstrates a statistically significant association between CT-based T staging and histopathological T staging (p < 0.001). Among the six patients with T2 disease on histopathology, CT correctly identified three (50%) cases, while the remaining three (50%) cases were overstaged as T3. Of the 14 patients with T3 disease on histopathology, CT accurately staged 12 cases (85.7%), whereas two cases (14.3%) were understaged as T2. The single patient with T4 disease (n = 1) was correctly identified as T4 on CT, resulting in 100% concordance in this category. These findings indicate that CT shows better agreement for advanced tumors (T3-T4) than for early-stage tumors (T2), particularly in the post-chemoradiation setting. Surgical T staging also showed significant concordance with histopathology (p < 0.001), especially for T3 and T4 lesions.

**Table 6:** T staging correlation of CT, surgery, and HPE staging. HPE: histopathological examination. * indicates statistical significance. A p-value less than 0.05 was considered statistically significant.

Parameters		T0		T1		T2		T3		T4		TX		Total		P-value
		N (%)	%	Count	%	Count	%	Count	%	Count	%	Count	%	Count	%	
CT T staging	T3	0	0.0%	0	0.0%	3	50.0%	12	85.7%	0	0.0%	0	0.0%	15	55.6%	<0.001*
	T4	0	0.0%	0	0.0%	0	0.0%	0	0.0%	1	100.0%	0	0.0%	1	3.7%	
	Tis-T2	2	100.0%	3	100.0%	3	50.0%	2	14.3%	0	0.0%	1	100.0%	11	40.7%	
Surgery T staging	T1	2	100.0%	2	66.7%	0	0.0%	0	0.0%	0	0.0%	0	0.0%	4	14.8%	<0.001*
	T2	0	0.0%	1	33.3%	6	100.0%	2	14.3%	0	0.0%	1	100.0%	10	37.0%	
	T3	0	0.0%	0	0.0%	0	0.0%	12	85.7%	0	0.0%	0	0.0%	12	44.4%	
	T4	0	0.0%	0	0.0%	0	0.0%	0	0.0%	1	100.0%	0	0.0%	1	3.7%	

Table 7 summarizes the diagnostic performance of CT in T staging using histopathology as the reference standard. For T2 tumors, CT demonstrated a sensitivity of 50.0%, specificity of 61.9%, positive predictive value of 27.3%, negative predictive value of 81.3%, and an overall diagnostic accuracy of 59.3%, reflecting modest performance in early-stage disease. In contrast, CT showed improved performance for T3 tumors, with a sensitivity of 85.7%, specificity of 76.9%, positive predictive value of 80.0%, negative predictive value of 83.3%, and overall accuracy of 81.5%. For T4 tumors, CT achieved 100% sensitivity, specificity, positive predictive value, negative predictive value, and diagnostic accuracy. However, this apparent perfect performance corresponds to a single T4 case and should therefore be interpreted cautiously, given the small sample size and wide confidence intervals. Overall, CT performance improved with increasing tumor stage.

**Table 7:** Validity of CT scan in T staging in comparison with HPE T staging. CI: confidence interval; HPE: histopathological examination. Confidence intervals were calculated using the exact (Clopper-Pearson) method. The wide confidence intervals observed for T4 parameters reflect the very small number of T4 cases (n = 1) and indicate limited precision of these estimates.

Diagnostic parameters	T2 stage (95% CI)	T3 stage (95% CI)	T4 stage (95% CI)
Sensitivity	50.0% (95% CI: 11.8-88.2)	85.71% (95% CI: 57.2-98.2)	100% (95% CI: 2.5-100)
Specificity	61.9% (95% CI: 38.4-81.9)	76.92% (95% CI: 46.2-95.0)	100% (95% CI: 86.8-100)
Positive predictive value	27.27% (95% CI: 6.0-61.0)	80.0% (95% CI: 51.9-95.7)	100% (95% CI: 2.5-100)
Negative predictive value	81.25% (95% CI: 54.4-96.0)	83.33% (95% CI: 51.6-97.9)	100% (95% CI: 86.8-100)
Diagnostic accuracy	59.26% (95% CI: 39.1-77.5)	81.48% (95% CI: 61.9-93.7)	100% (95% CI: 87.2-100)

Table 8 demonstrates a statistically significant association between CT N staging and histopathological N staging (p < 0.001). Of the 16 patients with N0 disease on histopathology, CT correctly identified 15 (93.8%) cases, while one (6.2%) case was overstaged. Among the four patients with N1 disease, CT accurately identified two (50%) cases, whereas two (50%) cases were misclassified. All cases classified as N2 on histopathology were correctly identified as advanced nodal disease on CT, showing complete agreement in this subgroup. Surgical nodal staging categorized 18 (66.7%) patients as node-negative and nine (33.3%) patients as node-positive intraoperatively. Among the surgically node-negative group, 14 (87.5%) cases were confirmed as N0 on histopathology, while four cases demonstrated nodal metastasis, indicating the limitations of intraoperative nodal assessment. These results suggest that CT performs well in excluding nodal metastasis and detecting higher nodal burden, but shows moderate sensitivity for limited nodal involvement.

**Table 8:** N staging correlation of CT, surgery, and HPE staging. HPE: histopathological examination. * indicates statistical significance. A p-value less than 0.05 was considered statistically significant.

Parameters		N0		N1		N2a		N2		NX		Total		p-value
		Count	%	Count	%	Count	%	Count	%	Count	%	Count	%	
CT N staging	N0	15	93.8%	1	25.0%	0	0.0%	0	0.0%	3	100.0%	19	70.4%	<0.001*
	N1	1	6.2%	2	50.0%	0	0.0%	0	0.0%	0	0.0%	3	11.1%	
	N2	0	0.0%	1	25.0%	3	100.0%	1	100.0%	0	0.0%	5	18.5%	
Surgery N staging	N-	14	87.5%	1	25.0%	0	0.0%	0	0.0%	3	100.0%	18	66.7%	0.003*
	N+	2	12.5%	3	75.0%	3	100.0%	1	100.0%	0	0.0%	9	33.3%	

Table 9 shows a statistically significant association between CT and histopathological M staging (p < 0.001). On CT evaluation, 23 (85.2%) patients were staged as M0 and four (14.8%) patients as M1. On histopathology, six (22.2%) patients were classified as M0, while 21 (77.8%) patients were categorized as Mx. CT demonstrated high concordance in identifying patients without distant metastasis. However, the large proportion of Mx cases on histopathology reflects the inherent limitation of surgical specimen pathology in determining distant metastatic status. These findings indicate that CT remains useful for detecting distant metastasis, although interpretation should consider the limited pathological confirmation in all cases.

**Table 9:** M staging correlation of CT, surgery, and HPE staging. HPE: histopathological examination. * indicates statistical significance. A p-value less than 0.05 was considered statistically significant.

Parameters		M0		M1		M1a		MX		Total		p-value
		Count	%	Count	%	Count	%	Count	%	Count	%	
CT M staging	M0	4	100.0%	1	100.0%	0	0.0%	20	95.2%	25	92.6%	<0.001*
	M1	0	0.0%	0	0.0%	0	0.0%	1	4.8%	1	3.7%	
	MX	0	0.0%	0	0.0%	1	100.0%	0	0.0%	1	3.7%	
Surgery M staging	M+	0	0.0%	0	0.0%	1	100.0%	0	0.0%	1	3.7%	<0.001*
	M0	4	100.0%	0	0.0%	0	0.0%	17	81.0%	21	77.8%	
	MX	0	0.0%	1	100.0%	0	0.0%	4	19.0%	5	18.5%	

Overall, there was a strong correlation between CT, surgical, and histopathological staging. Concordance rates were 20 (74.1%) for T-staging, 24 (88.9%) for N-staging, and 25 (92.6%) for M-staging (Table 10).

**Table 10:** Correlation of T stage, N stage, and M stage.

Parameters		Count	%
Correlation T staging	No	7	25.9%
	Yes	20	74.1%
Correlation N staging	No	3	11.1%
	Yes	24	88.9%
Correlation M staging	No	2	7.4%
	Yes	25	92.6%

## Discussion

The present study aimed to evaluate CT accuracy in staging rectal carcinoma in patients who had received neoadjuvant CRT and to compare CT findings with operative and histopathological results, with additional assessment of CT performance in evaluating lymph node status. Rectal carcinoma was initially staged on CT, subsequently staged intraoperatively, and final staging was determined by histopathological examination. Colonoscopy with histopathological confirmation preceded CT evaluation in all cases. In a colonoscopy-based study of 220 patients, Peedikayil et al. [9] reported that nearly one-third of colorectal cancers occurred during the fifth to sixth decades of life, approximately a decade earlier than in Western populations, and that most tumors were distal to the splenic flexure. In the present study, patient ages ranged from 29 to 80 years, with most falling within the 61-70-year age group. There was a female predominance (16 females, 59.3%; 11 males, 40.7%), and preoperative anemia was observed in 21 of 27 patients (77.8%), higher than the 44% reported by Mohandas [10].

Previous studies have shown that CT accurately detects rectal and rectosigmoid carcinoma, with Thoeni et al. [11] reporting 100% sensitivity and overall staging accuracy of 95%. Chiesura-Corona et al. [8] identified CT as the preferred modality for staging due to its availability and standardization, reporting sensitivities of 81.9% for Tis-T2 lesions, 82.5% for T3 lesions, and 75% for T4 lesions, noting some overstaging and understaging across T stages. Although MRI remains superior for local staging, CT remains widely used because it is accessible, cost-effective, time-efficient, and reliable for detecting distant metastases. Its limitations include lower soft-tissue resolution and challenges in differentiating T1 from T2 lesions, with distinction between T2 and T3 tumors critical for management, as T3 lesions are typically identified on MDCT by perirectal fat stranding.

Although MRI is widely regarded as the preferred modality for detailed local staging and post-CRT restaging due to its superior soft-tissue resolution, CT was utilized in the present study because of its greater availability, lower cost, shorter acquisition time, and integration into the routine oncologic workflow at our institution. In many resource-constrained settings, MRI access may be limited or associated with scheduling delays, whereas multidetector CT is readily accessible and routinely performed for systemic staging. Additionally, CT provides simultaneous evaluation of the primary tumor, regional lymph nodes, liver, lungs, and peritoneum in a single examination, which is particularly valuable in post-CRT patients where assessment of metastatic disease and resectability is critical [7-9]. CT findings in our study directly influenced surgical planning by identifying advanced T-stage disease (T3-T4), assessing adjacent organ involvement, evaluating nodal burden, and detecting distant metastases that may alter operability or necessitate modification of treatment strategy.

In our study, CT correctly staged 12 T3 tumors, while two T2 lesions were understaged as Tis-T2 due to absent perirectal fat stranding, and one T4 lesion was correctly staged on both CT and histopathology. Sensitivity for T2, T3, and T4 lesions was 50.0%, 85.7%, and 100.0%, respectively, with corresponding specificities of 61.9%, 76.9%, and 100.0%. Positive predictive values were 27.3%, 80.0%, and 100.0%, and negative predictive values were 81.3%, 83.3%, and 100.0%, resulting in overall diagnostic accuracies of 59.3%, 81.5%, and 100.0% for T2, T3, and T4 lesions, indicating improved CT performance with advancing T stage.

Lymph node staging is crucial for diagnosis, prognostication, and management planning in rectal cancer. CT demonstrated strong diagnostic performance for T3 lesions, with 85.7% sensitivity and 76.9% specificity, showing substantial agreement with surgical and histopathological findings across T, N, and M categories. These results align with Regge et al. [12], who reported 84% overall accuracy, and Choi et al. [13], who observed reviewer-dependent variability in nodal staging with sensitivity ranging from 54% to 88%, specificity from 58% to 66%, and overall accuracy of 70%. Singla et al. [14] similarly reported the rectum as the predominant site of involvement, demonstrating CT sensitivities of 83.3% for T1-T2, 88.2% for T3, and 100% for T4 lesions with excellent specificity.

Our findings are further supported by recent literature. A 2024 study [15] reported good CT sensitivity, specificity, and accuracy compared with MRI, particularly in evaluating tumor stage, mesorectal fascia involvement, and extramural vascular invasion. Ujala et al. [16] observed high diagnostic performance of perfusion CT, with 90.3% sensitivity, 88.6% specificity, and 89.6% accuracy. Alçın et al. [17] demonstrated that locoregional nodal maximum standardized uptake value (SUVmax) on PET/CT significantly influenced prognosis, underscoring the prognostic value of imaging. While MRI remains central for precise local staging and post-CRT restaging [18-22], CT remains invaluable for advanced disease, rapid assessment, and evaluation of distant metastases. Functional imaging, including quantitative apparent diffusion coefficient (ADC) mapping, has also been shown to improve nodal characterization and treatment planning [20]. Furthermore, Kim et al. [21] reported that lymph node ratio is a stronger prognostic marker than conventional N staging for predicting recurrence and survival, emphasizing the integration of radiological accuracy with prognostic markers to enhance risk stratification.

The present study highlights the diagnostic efficacy of CT in staging advanced rectal carcinoma (T3-T4), consistent with previous literature emphasizing its role in assessing metastatic disease and acute complications. At the same time, MRI remains the modality of choice for detailed local evaluation. Although diffusion-weighted MRI and other functional imaging techniques provide additional benefits for post-CRT restaging and organ-preserving strategies, our findings confirm that CT is a reliable, widely available, and cost-effective modality for staging advanced rectal carcinoma and identifying distant metastases.

Limitations

This study has several limitations. The sample size was relatively small (n = 27) compared with larger cohorts, which may limit statistical power, subgroup analysis, and generalizability of the findings. Although diagnostic parameters were calculated with 95% confidence intervals, the small cohort size may have resulted in wider intervals and reduced precision of the estimates. Furthermore, the single-center design may limit the external validity of the results. Additionally, the interval between CRT, CT evaluation, and surgery was considerable, resulting in some loss to follow-up and potential bias in the final analysis. The lack of uniform pathological confirmation of distant metastases in all patients limited M staging assessment. A significant proportion of cases were categorized as Mx on histopathology, which may reduce the validity of CT-histopathology comparison for distant metastasis.

## Conclusions

In conclusion, MDCT demonstrates moderate-to-high diagnostic performance in post-chemoradiation restaging of rectal carcinoma, particularly in advanced T-stage disease (T3-T4). Its accuracy was comparatively lower for early-stage tumors (T2), reflecting known limitations of CT in differentiating mural layers after chemoradiation. MDCT remains useful for assessing nodal involvement and distant metastasis, especially in evaluating advanced disease burden and surgical planning. However, given the small sample size and limited subgroup numbers in this study, these findings should be interpreted cautiously. MRI remains the preferred modality for detailed local staging and early-stage tumor assessment.
